# A Case of Initially Undiagnosed Chikungunya Arthritis Developing into Chronic Phase in a Nonendemic Area

**DOI:** 10.1155/2017/2592964

**Published:** 2017-03-20

**Authors:** Pui Shan Julia Chan, Moon Ho Leung

**Affiliations:** Department of Medicine, Queen Elizabeth Hospital, Kowloon, Hong Kong

## Abstract

This case report described a 40-year-old lady presented with fever, headache, arthralgia, myalgia, and impaired liver function after returning from the Philippines. Chikungunya virus (CHIKV) and dengue serology were negative. Eight weeks after initial presentation, she experienced inflammatory polyarthritis mimic rheumatoid arthritis. This time CHIKV-IgM was detected, together with a >4-fold rise of CHIKV-polyvalent-antibody titre. The first CHIKV-IgM negative sample was reexamined and was CHIKV-PCR positive. CHIKV infection was confirmed and diagnosis of CHIKV-related arthritis was made. A quarter of CHIKV infected individuals develop post-CHIKV rheumatisms that affect quality of life and may need treatment with Disease Modifying Antirheumatic Drugs. This case highlights the importance of considering CHIKV infection in patients present with symmetrical polyarthritis particularly after travel to endemic regions. Testing of both CHIKV acute and convalescent-phase serum for CHIKV antibodies and PCR is recommended in suspicious case.

## 1. Introduction

Chikungunya fever is a mosquito-borne disease. Diagnosis can be challenging as most of the infected individuals present with nonspecific symptoms. We report a case of initial CHIKV-IgM negative patient, later developed symmetrical polyarthritis resembles rheumatoid arthritis (RA), and CHIKV serology turned positive 8 weeks later. Convalescent-phase serum should always be obtained from patients whose acute-phase samples test is negative.

## 2. Case Presentation

A 40-year-old lady presented with fever, headache, arthralgia, and myalgia, after returning from the Philippines. She recalled mosquito bite one week before. Investigations showed lymphopenia, increased hepatic enzymes, C-Reactive-Protein (CRP), normal renal function (Table 1), and prominent spleen (11.6 cm) on ultrasonogram. Empirical antibiotic was given. Septic workup was negative. Her fever subsided and hepatic enzymes were improving. Dengue-virus IgM/specific nonstructural (NS1) protein antigen, chikungunya virus (CHIKV) IgM, and blood malaria parasite were not detected. She was discharged on day 10 with stable condition.

**Figure 1:**
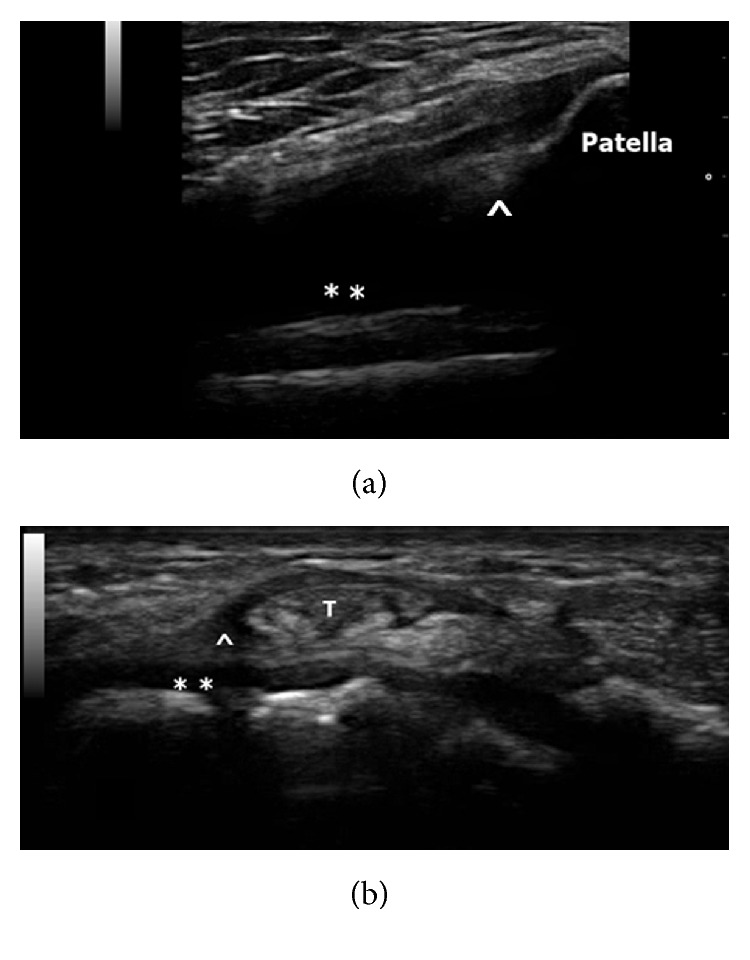
Ultrasound of knee and wrist. (a) Longitudinal scan of knee demonstrated hypoechoic joint fluid (*∗∗*) and synovial hypertrophy (∧) at suprapatellar recess. (b) Transverse scan of dorsum of wrist showed hypoechoic effusion (*∗∗*) and rim of fluid compatible with tenosynovitis (∧) of extensor tendon (T).

Eight weeks later she was readmitted for fever, malaise, and persistent symmetrical polyarthritis involving both wrists, metacarpophalangeal joints, shoulders, hips, and knees, mimicking RA. She reported no recent hiking, no rash, and no oral ulcer. Clinical examination revealed more than ten tender and swollen joints over hands, wrists, and knees. There was no lymphadenopathy and no focal neurological deficit. Blood tests showed elevated liver enzymes, ferritin, and persistent high CRP level (Table 1). Hepatitis A, B, and C serology, anti-HIV, autoantibodies including rheumatoid factor, anticyclic-citrullinated peptide, anti-nuclear antibody, antidouble-stranded-DNA, anti-neutrophil-cytoplasmic antibody, anti-smooth-muscle antibody, and anti-mitochondrial antibody were all negative. C3 and C4 levels were normal. There was no erosion on hands and knees radiographs and her chest X-ray was clear. She was covered with Augmentin and Doxycycline for fever. No vegetation was detected on echocardiogram. Hepatic ultrasonogram showed prominent periportal echogenicity. Musculoskeletal ultrasound demonstrated wrists extensors and flexors tenosynovitis with increased Doppler signals, effusion, and synovial proliferation in knees (Figure 1). A total 25 mL fluid was aspirated from left knee suprapatellar recess. Gram stain, bacterial culture, and acid-fast-bacilli smear were negative. Fever persisted for two weeks. Liver biopsy was suggested to look for infective or infiltrative causes but patient refused. Septic workup was all unrevealing. CHIKV serology was rechecked. This time CHIKV-IgM was detected and CHIKV-polyvalent-antibody titre had risen from 1 : <10 to 1 : 1280. The first CHIKV-IgM negative sample was reexamined and was CHIKV-PCR positive (Table 1). The diagnosis of CHIKV-related arthritis was made. She was treated with supportive measures. Liver enzymes slowly reduced but joint symptoms persisted. Patient requested discharge-against-medical-advice after 3 weeks of hospital stay.

## 3. Discussion

Chikungunya fever is a mosquito-borne disease, endemic to Africa and Southeast Asia, caused by CHIKV, a single-stranded RNA virus that belongs to the family Togaviridae, genus* Alphavirus*. Differential diagnosis of CHIKV infection includes dengue fever, West Nile fever, Zika virus infection, and other common infections like parvovirus, rubella, and malaria. At the 2004–2011 CHIKV epidemic, 1.4 to 6.5 million individuals in over 40 countries have been affected by the virus [1]. Coinfection of dengue and CHIKV has been reported, where concurrent dengue and CHIKV outbreaks occurred. At present, no vaccine or efficient treatment is available for CHIKV infection and preventive measures play a crucial role.

Symptoms of CHIKV infection are nonspecific. Common manifestations are fever, arthralgia, fatigue, and rash. Rarer symptoms include neuroretinitis, myocarditis, pericarditis, pneumonia, vasculitis, nephritis, fulminant hepatitis, pancreatitis, and neurological involvement [2, 3]. Although most cases are self-limiting or even asymptomatic, deaths have been reported in patients with cardiovascular, renal, hepatic, and nervous system involvement. In the acute phase of infection, high viraemic load with a median duration of viraemia of 6 days (range 3–10 days) and concomitant lymphopenia and/or thrombocytopenia is typical of CHIKV infection [2]. Antibodies to CHIKV normally develop toward the end of the first week of illness. In this patient, the initial serum checked one week after mosquito bite was CHIK-IgM negative but turned positive 8 weeks later. CHIKV infection in the first week can be IgM negative and only PCR positive. Therefore CHIKV acute and convalescent-phase serum should be obtained for patients with compatible symptoms and acute-phase samples test negative.

Because of the prolonged joint symptoms, CHIKV-related arthritis can be mistaken as seronegative RA, whereas such symptoms are uncommon in dengue. The pathogenesis of CHIKV-related arthritis is poorly understood. CHIKV has not been cultured from synovial fluid, but viral RNA can be detected in the synovium, suggesting that CHIKV may directly invade and persist in joints. Overlapping clinical and immunologic evidence between CHIKV and RA has been reported. In a cohort of CHIKV infected patients, cytometry analysis revealed that these patients, when compared to RA patients, had similar natural killer and T-cell profiles including similar percentages of naive, activated, and effector T killer and helper T-cells. Moreover, CHIKV infected and RA patients had greater percentages of activated and effector CD4+ and CD8+ T-cells than healthy controls [4].

CHIKV infection is recognized to be a two-stage disease. After the short-lived improvement following the acute stage due to viraemia, patients might experience a rebound of general discomfort, exacerbation of inflammatory, relapses, long-lasting rheumatism, and an increased handicap in daily life [5]. The link between CHIKV and chronic inflammatory rheumatisms (CIR) has become increasingly reported and is suggested to be an immune-mediated condition. Any polyarticular inflammatory feature persisting more than 3 months after CHIKV infection must suggest the potential for a diagnosis of post-CHIK-CIR. Two main categories of chronic CHIKV disease have been proposed: post-CHIKV musculoskeletal disorders and post-CHIKV de novo CIR. The former have been managed with analgesics, anti-inflammatory drugs, and physiotherapy, whereas post-CHIKV de novo CIR includes RA, spondyloarthritis, and undifferentiated polyarthritis which occur in patients without history of rheumatic disease before CHIKV infection [6]. In a recent systemic review, 25% of CHIKV infected patients suffered from post-CHIKV rheumatisms, having either arthralgia, musculoskeletal pain, or arthritis and 14% developed chronic arthritis. Erosive forms have reported that some patients required Disease Modifying Antirheumatic Drugs [7]. Thus post-CHIKV CIR may pose a burden on public health if CHIKV infection is not adequately addressed and controlled. Patients should be advised to use barrier method to prevent sexual transmission.

The optimal treatment of post-CHIKV arthritis is not clear. Intensive therapy with Disease Modifying Antirheumatic Drugs (DMARD) or biologics has been reported [6, 8]. A recent study which compared the efficacy of triple therapy (hydroxychloroquine, sulphasalazine, and methotrexate) with hydroxychloroquine alone showed a significant improvement in disease activity and disability in the triple therapy group at 24 weeks [9].

In summary, we reported a case of post-CHIKV-related polyarthritis. As an emerging disease, high clinical vigilance, and checking of CHIKV acute and convalescent-phase serum for CHIKV antibodies and PCR should be performed in indicated case. Rheumatology opinion should be considered for patients who developed chronic arthritis as initiation of DMARD may be necessary.

## Figures and Tables

**Table 1:** Summary of laboratory investigations.

Investigations	First presentation	8 weeks later	Normal range
Haemoglobin (g/dL)	14.1	13.2	11.7–14.9
White cell count (×10^9^/L)	5.6	11.3	3.7–9.2
Lymphocytes (×10^9^/L)	0.7	2.0	1.0–3.1
Neutrophils (×10^9^/L)	4.4	8.4	1.7–5.8
Platelets (×10^9^/L)	256	435	145–370
C-reactive protein (mg/L)	33	142	<5
GGT (IU/L)	258	383	6–42
ALP (IU/L)	143	391	34–79
ALT (IU/L)	109	326	8–36
AST (IU/L)	95	212	<32
Ferritin (*ρ*mol/L)	345	993	14–535
Total protein (*μ*mol/L)	80	80	66–80
Total bilirubin (*μ*mol/L)	7	11	5–27
Urea (*μ*mol/L)	2.8	4.1	3.0–7.4
Creatinine (*μ*mol/L)	48	40	47–82
CHIKV IgM	Negative	Positive	—
CHIKV polyvalent antibody	1 : <10	1 : 1280	—
CHIKV-PCR	Positive (reexamined)	—	—
